# Congenital Hemifacial Hyperplasia: Clinical Presentation and Literature Review

**DOI:** 10.1155/2016/5260645

**Published:** 2016-10-24

**Authors:** Karpagavalli Shanmugasundaram, V. K. Vaishnavi Vedam, Sivadas Ganapathy, Sivan Sathish, Parvathi Satti

**Affiliations:** ^1^Department of Oral Medicine & Radiology, Saveetha Dental College, Saveetha University, Chennai, India; ^2^Department of Oral Pathology, Faculty of Dentistry, Asian Institute of Medicine, Science & Technology (AIMST) University, Kedah, Malaysia; ^3^Department of Pedodontics and Preventive Dentistry, Faculty of Dentistry, Asian Institute of Medicine, Science & Technology (AIMST) University, Kedah, Malaysia; ^4^Department of Oral Medicine and Radiology, Chettinad Dental College & Research Institute, Kancheepuram, India; ^5^Chettinad Dental College & Research Institute, Kancheepuram, India

## Abstract

Hemifacial hyperplasia is a rare congenital malformation characterized by noticeable unilateral excess development of hard and soft tissues of the face. Asymmetry in Congenital Hemifacial Hyperplasia (CHH) is usually evident at birth and accentuated at the age of puberty. The affected side grows exponentially as compared to the unaffected side. Multiple tissue involvement has resulted due to etiological heterogeneity like heredity, chromosomal abnormalities, altered intrauterine environment, and endocrine dysfunctions. As this lesion is rarely seen in our routine clinical practice, we present a case of hemifacial hyperplasia with reported orofacial features that supplement existing clinical knowledge. This paper also adds knowledge to the readers regarding detailed investigation procedures which has complemented our diagnosis. Further emphasis has been placed on periodic approach to its diagnosis and multidisciplinary management following correct diagnosis.

## 1. Introduction

Congenital Hemifacial Hyperplasia (CHH) is a rare developmental malformation exhibited by a unilateral enlargement of hard and soft tissues of the face. This asymmetrical overgrowth traditionally referred to as hypertrophy is more accurately termed as hyperplasia as this pathologic process involves abnormal proliferation of cells (hyperplasia) rather than growth of individual existing cells (hypertrophy) [1]. Synonymously, this condition has been termed as facial hemihyperplasia, partial/unilateral gigantism, hemimacrosomia, and hemifacial hyperplasia [2].

Congenital Hemifacial Hyperplasia was first noted by Meckel JF in 1822 and, later, first case was reported by Wagner in 1839 [3]. Later, Gesell described this lesion as being “essentially a developmental anomaly antedating birth and arising in partial deflection of the normal process of birth” [4].

Hyperplasia presents as an isolated finding or in association with several syndromes like Beckwith Wiedemann syndrome, Proteus syndrome, Russell silver syndrome, and Sotos syndrome. Rowe [5] proposed a classification of hemihyperplasia based on its anatomic location as (1) simple hemihyperplasia (single limb), (2) complex hemihyperplasia (one-half of the body), and (3) facial hemihyperplasia (one side of the face). Depending on the soft tissue involvement, hemifacial hyperplasia can be broadly classified as (1) true hemifacial hyperplasia (increased growth of one or more tissues on one side of the face) and (2) partial hemifacial hyperplasia (increased growth limited to one structure only) [6].

We present an interesting case of true congenital hemifacial hyperplasia (true CHH) in a 39-year-old male with unique orofacial manifestations, radiographic findings, and differential diagnosis with treatment in detail to supplement the existing knowledge.

## 2. Case Presentation 

A thirty-nine-year-old male patient reported to the Department of Oral Medicine and Radiology with a chief complaint of asymptomatic swelling in his right cheek region since birth. The swelling gradually progressed to the present size and ceased to grow after 18 yrs of age. Family history was noncontributory. The patient was well oriented with stable vital signs.

**Figure 1:**
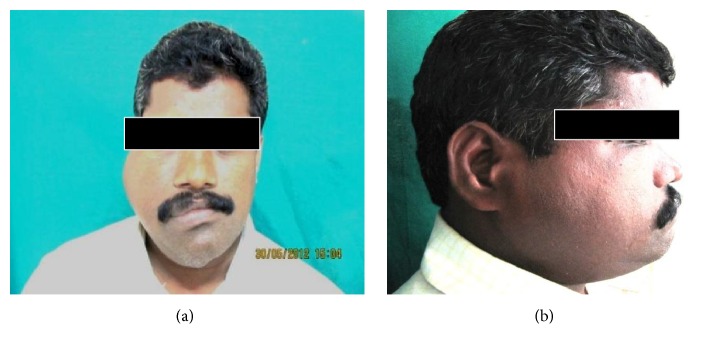
((a) and (b)) Extraoral examination of the patient exhibiting a diffuse swelling pertaining to hard and soft tissues of right side of the face.

On extraoral examination, facial asymmetry with diffuse swelling was evident on the right side of the face measuring about 5 × 6 cm. The swelling extended superiorly to upper canthus of right eye, inferiorly up to 1 cm below the lower border of mandible, anteriorly until nasolabial fold, and posteriorly till the tragus of the ear [Figures 1(a) and 1(b)]. Nose and chin were deviated with an observable arc shaped facial midlines (nasion-gnathion). Enlarged soft tissue mass was observed involving maxilla, mandible, and zygoma on the affected side. On palpation swelling was nontender, hard in consistency, and noncompressible. Skin over the swelling was normal with no evidence of secondary changes. Patient showed mild tenderness on the right condylar region. Restricted temporomandibular joint (TMJ) movements and incompetent lips with marked enlargement of both upper and lower lips on the right side were evident. There was no evidence of regional lymphadenopathy.

**Figure 2:**
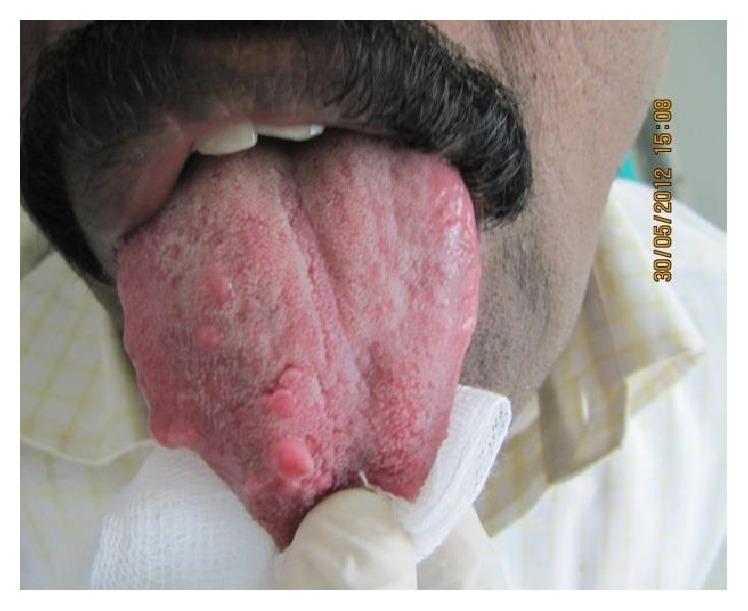
Intraoral examination of dorsum surface of the tongue showing multiple nodular structures exhibiting “pebbly” appearance on the right side only.

**Figure 3:**
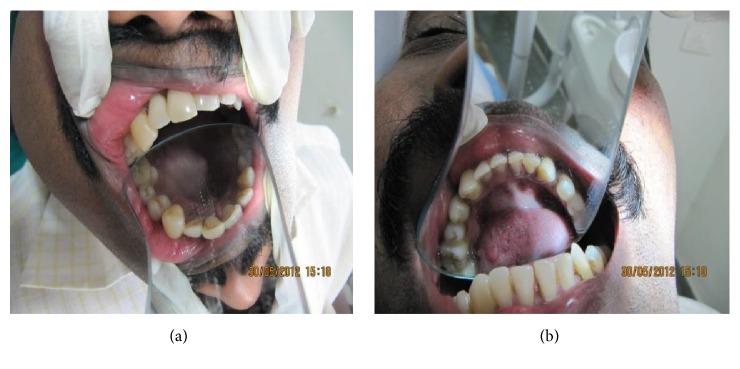
((a) and (b)) Intraoral examination showing right sided enlargement of maxillary and mandibular arches with corresponding macrodontia.

On intraoral examination, there was restricted mouth opening with a maximum interincisal distance measuring about 1 cm only. Enlarged right maxillary and mandibular alveolar arches, upper and lower labial mucosa, and buccal mucosa were observed. Dorsum surface of the tongue appeared engorged with polypoid excrescences (“multiple pebbly” appearance) representing enlargement of fungiform papillae [Figure 2]. Distinct tooth size discrepancy was evident between right and left sides. Maxillary and mandibular right teeth increased in labiolingual, mesiodistal, and buccopalatal dimensions [Figures 3(a) and 3(b)]. Midline shift and a downward canting of the occlusal plane were noted.

**Figure 4:**
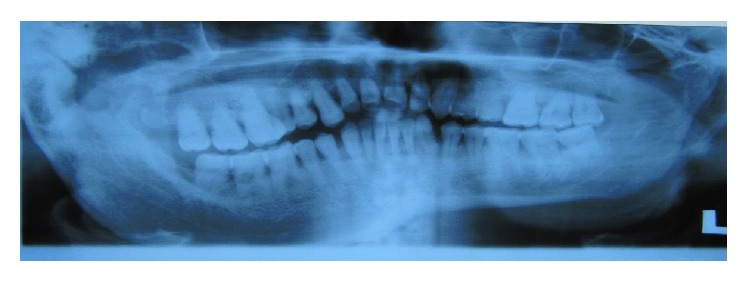
OPG reveals diffuse enlargement of skeletal and dental hard tissue enlargement on right side of the face.

**Figure 5:**
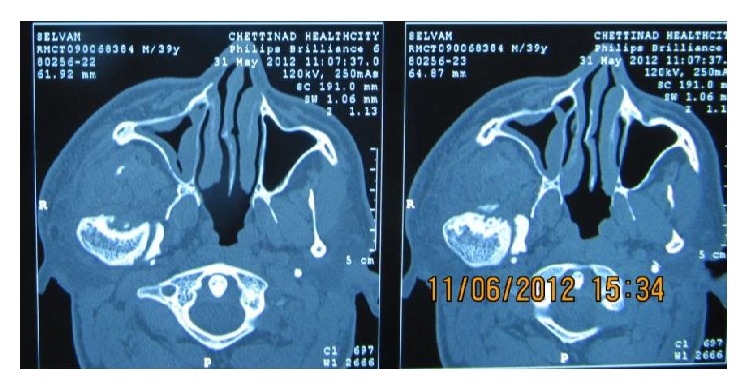
CT scan exhibits enlarged petrous part of temporal bone, pituitary fossa, maxilla, and mandible.

**Figure 6:**
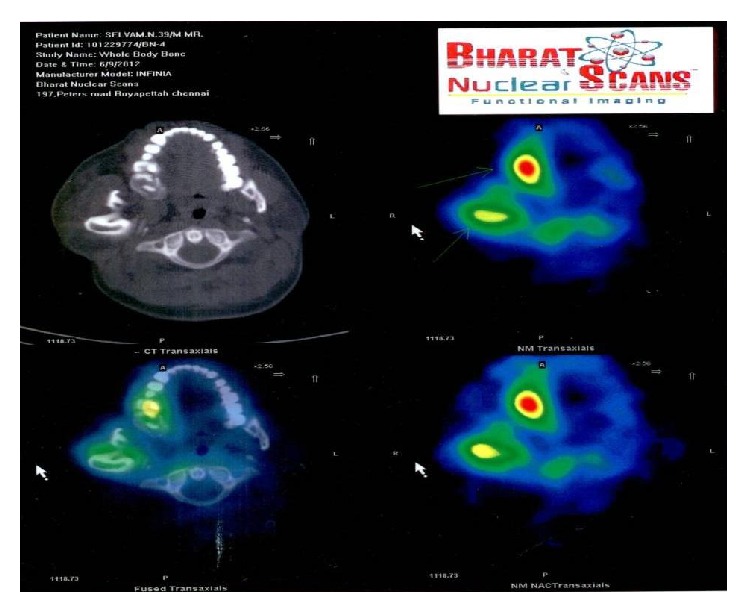
Bone scintigraphy shows increased uptake and hyperactivity in right sided maxilla and mandible of the face.

Orthopantomogram (OPG) [Figure 4] revealed an obvious diffuse enlargement of right side coronoid, condylar processes, lower border of mandible, inferior alveolar canal, jaws, and teeth (macrodontia). Computerized tomography (CT) [Figure 5] scan of the face revealed enlarged right petrous part of temporal bone, pituitary fossa, maxilla, mandible, condyle, zygoma, and cranial bones. Deviation of nasal bone and chin was observed towards left side due to an obvious enlargement of overlying soft tissues on right side of the face. Bone scintigraphy [Figure 6] revealed hyperactivity and excess bone growth on right side maxilla and mandible. A physician consultation was arranged and no systemic abnormality was noted. Routine blood investigations were also under normal limits.

Based on the clinicopathological findings, a final diagnosis of Congenital Hemifacial Hyperplasia (CHH) was made. The patient refused to undergo extensive surgical procedures as the lesion was asymptomatic. Follow-up with this patient exhibited good prognosis and no evidence of malignant changes.

## 3. Discussion

Hemifacial hyperplasia (HFH) is a rare developmental anomaly exhibiting asymmetric facial growth by unilateral localized enlargement of all tissues in the affected area that is facial bones, teeth, and soft tissues [5]. The degree of anatomical variations appears variable from mild to most severe cases. Hence limited cases of hemifacial hyperplasia are documented.

The prevalence of hemifacial hyperplasia is approximately 1 in 86,000 live births [1, 3]. Women are commonly affected compared to men with right side predominantly affected as in the present case. The affected side tissue growth occurs in an exponential manner in comparison to the uninvolved side resulting in facial asymmetry following a skeletal arrest. This results in a relative asymmetry that is taken forward throughout the growth until adulthood [7].

Various etiological factors like heredity, chromosomal abnormalities, altered intrauterine development, endocrine dysfunction, and vascular and lymphatic abnormalities remain implicated in this lesion. However, no single factor is directly related to the occurrence of this disorder [8].

Of importance, Gesell suggested this condition due to the process of twinning mechanism. According to Noe and Bergman, mitochondrial damage overripened one-half of the fertilized egg resulting in excess generation of cells [9]. Recently, literature suggests that hemifacial hyperplasia may be more aptly attributed to defect in neural crest cell differentiation and migration. In this theory, Pollock and colleagues stated that neural fold appears to be larger on one side compared to the other part of structure. This enlarged neural fold produces increased number of neural crest cells throughout prenatal and postnatal periods of life owing to unilateral overgrowth of crest cell derived bone, teeth, and soft tissue on affected side of the face. Based on the current knowledge, Yoshimoto et al. stated that basic fibroblast growth factor (bFGF) along with its receptor stimulated osteoblastic differentiation on the affected side as compared to the uninvolved part of the face [10].

Limited cases of hemifacial hyperplasia have been documented. These patients present with dental, skeletal, and soft tissues of the affected portion of the face [11]. Dental abnormalities of deciduous teeth are less common and often overlooked. Variations in size, shape of crowns and root of teeth, rate of development (precocious development), and number of teeth are seen. Tooth crown and root size/shape appear distorted. Permanent canines, bicuspids, and first molars are involved most frequently [12, 13]. Of note, our case presented with marked dental abnormalities like precocious tooth development and macrodontia.

Skeletal development of cranial bones* (frontal, parietal, temporal, and skull base)*, zygoma, maxilla, and mandible is accentuated. Midline shift and altered occlusion with deviated occlusal plane are noticed. Accelerated jaw growth frequently results in exostoses with malocclusion [14, 15]. Soft tissue abnormalities include gingival and labial mucosal thickening. Large soft tissue excrescences are evident predominantly on the dorsum of tongue (“pebbly”) and buccal mucosa (“lipoma-like growth”). Tongue exhibits enlargement of fungiform papillae unilaterally. Lower lip is twice commonly affected than upper lip. Involvement of upper lip causes displacement of philtrum to the uninvolved side of the face. Palate also shows an arch shaped deformity ipsilaterally [16, 17]. All these skeletal features and soft tissue abnormalities were evident in the above case.

Radiographically, this lesion presents with hard tissue and soft tissue enlargement on the affected side. These patients exhibit premature development and altered crown and root size/shape discrepancy along with a downward slant of the occlusal plane [18, 19]. Our case is consistent with the previous findings.

Differential diagnosis of facial hemihyperplasia includes fibrous dysplasia, dyschondroplasia, congenital lymph edema, arteriovenous aneurysm, hemangioma, lymphangioma, neurofibromatosis, and malignant lesions (osteosarcoma and chondrosarcoma). These clinical conditions can be clearly differentiated from CHH based on specific radiographs and laboratory and clinical findings that supplemented our case diagnosis [20].

Treatment ideally is not indicated for Congenital Hemifacial Hyperplasia (CHH) unless cosmetic considerations are involved. Procedures are deferred until physiological growth ceases. An integrated multidisciplinary approach in the field of dentistry is essential in providing an asymptomatic treatment. Treatment options may include soft tissue debulking of masticatory and subcutaneous tissues, preservation of neuromuscular functions, and reconstructive procedures (osteotomy/orthognathic surgical procedure with/without orthodontic treatment) postoperatively. Till date, previous literature survey indicated no evidence of malignant transformation.

## 4. Conclusion 

A true case of Congenital Hemifacial Hyperplasia (CHH) is rare and unfamiliar to a clinician due to underreported number of cases in literature. This article presents a unique case report of a typical case of CHH with a detailed emphasis on diagnostic criteria (clinical and radiographic appearance) thus emphasizing to all the readers its importance. Treatment planning in such patients is tremendously difficult due to the involvement of multiple major surgeries for the removal of excessively grown hard and soft tissues. Further cases need to be brought into documentation so as to have a clear understanding regarding various aspects of this disease.
